# Geochemistry and Mixing Drive the Spatial Distribution of Free-Living Archaea and Bacteria in Yellowstone Lake

**DOI:** 10.3389/fmicb.2016.00210

**Published:** 2016-02-29

**Authors:** Jinjun Kan, Scott Clingenpeel, Charles L. Dow, Timothy R. McDermott, Richard E. Macur, William P. Inskeep, Kenneth H. Nealson

**Affiliations:** ^1^Stroud Water Research Center, AvondalePA, USA; ^2^DOE Joint Genome Institute, Walnut CreekCA, USA; ^3^Department of Land Resources and Environmental Sciences, Montana State University, BozemanMT, USA; ^4^Department of Earth Sciences, University of Southern California, Los AngelesCA, USA

**Keywords:** Yellowstone Lake, Bacteria and Archaea, pyrosequencing, spatial distribution, geochemistry

## Abstract

Yellowstone Lake, the largest subalpine lake in the United States, harbors great novelty and diversity of Bacteria and Archaea. Size-fractionated water samples (0.1–0.8, 0.8–3.0, and 3.0–20 μm) were collected from surface photic zone, deep mixing zone, and vent fluids at different locations in the lake by using a remotely operated vehicle (ROV). Quantification with real-time PCR indicated that Bacteria dominated free-living microorganisms with Bacteria/Archaea ratios ranging from 4037:1 (surface water) to 25:1 (vent water). Microbial population structures (both Bacteria and Archaea) were assessed using 454-FLX sequencing with a total of 662,302 pyrosequencing reads for V1 and V2 regions of 16S rRNA genes. Non-metric multidimensional scaling (NMDS) analyses indicated that strong spatial distribution patterns existed from surface to deep vents for free-living Archaea and Bacteria in the lake. Along with pH, major vent-associated geochemical constituents including CH_4_, CO_2_, H_2_, DIC (dissolved inorganic carbon), DOC (dissolved organic carbon), SO_4_^2-^, O_2_ and metals were likely the major drivers for microbial population structures, however, mixing events occurring in the lake also impacted the distribution patterns. Distinct Bacteria and Archaea were present among size fractions, and bigger size fractions included particle-associated microbes (> 3 μm) and contained higher predicted operational taxonomic unit richness and microbial diversities (genus level) than free-living ones (<0.8 μm). Our study represents the first attempt at addressing the spatial distribution of Bacteria and Archaea in Yellowstone Lake, and our results highlight the variable contribution of Archaea and Bacteria to the hydrogeochemical-relevant metabolism of hydrogen, carbon, nitrogen, and sulfur.

## Introduction

Microorganisms are the foundation of aquatic food webs, with heterotrophic microorganisms acting as fundamental consumers mediating organic matter mineralization, thus playing key roles in nutrient biogeochemical cycling (Carlsson and Caron, 2001; Salcher et al., 2010). Monitoring the distribution of these microorganisms and investigating the potential environmental drivers of this distribution are important to our understanding of the roles of these microbes and the integral functionality they contribute to aquatic ecosystems such as lakes. Depending on the morphology and geological features, bigger lakes are often characterized by strong environmental gradients in physicochemical parameters including temperature, salinity, oxygen, nitrogen, etc. These environmental gradients result in niche separation and differentiation, leading to the structuring and distribution of distinct microorganisms at different layers within lakes. Cole et al. (1993) found bigger and more abundant cells present in anoxic hypolimnia than oxic conditions. Later studies with molecular approaches confirmed this observation and led to the discovery of certain groups of microbes that preferentially inhabit deeper lake layers such as fermenting bacteria, denitrifying bacteria, methylotrophs, autotrophic sulfur bacteria, and Bacteroidetes (Casamayor et al., 2000; Lehours et al., 2007; Salcher et al., 2008, 2010). In general, microbial distribution patterns were likely responding to environmental gradients including oxygen (Cole et al., 1993; Lehours et al., 2007; Salcher et al., 2008, 2010), nutrients (Cole et al., 1993; Casamayor et al., 2000), and the geochemical characteristics of the lakes, such as salinity in hyper-saline environments (Baricz et al., 2014). However, due to complex environmental scenarios in lakes, monitoring microbial distribution and clarifying their environmental drivers still remains a challenge in aquatic microbial ecology.

**FIGURE 1:**
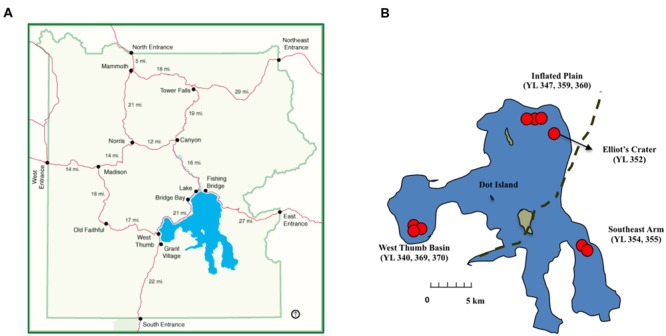
**Maps illustrating the relative locations of the research and sampling sites described in this study. (A)** Yellowstone National Park (downloaded from http://www.nps.gov/features/yell/interactivemap/index.htm) and **(B)** Yellowstone Lake (adapted from Clingenpeel et al., 2011 with permission). Red dots provided approximate lake locations for the nine sampling sites at West Thumb Basin, Inflated Plain, Elliot’s Crater, and Southeast Arm. Dashed line represent the approximate boundary of the Yellowstone caldera.

Yellowstone Lake (YL), the largest (∼352 km^2^) sub-alpine high-altitude lake in North America is a pristine, non-regulated body of water with a long (10-year) retention time (Benson, 1961; Morgan et al., 2007). The lake is critical to the function of the Yellowstone ecosystem (Schullery and Varley, 1995), and it contributes approximately 10% of the total geothermal flux in Yellowstone National Park (YNP; Balistrieri et al., 2007). Hundreds of lake floor vent features have been documented in the Yellowstone Lake by employing bathymetric, seismic, and submersible remotely operated vehicle (ROV) equipment (Morgan et al., 2003, 2007; Balistrieri et al., 2007). These vents occur primarily in the northern and West Thumb regions of the lake, although a relatively minor vent area occurs in the proximity of Dot Island (**Figure 1**). By mixing with the lake water, these strong geochemical signatures provide numerous niches capable of supporting phylogenetically and functionally diverse microbial populations. Recently, considerably novel and diverse populations of Bacteria, and Archaea were observed in the Yellowstone Lake (Clingenpeel et al., 2011, 2013; Kan et al., 2011; Yang et al., 2011; Inskeep et al., 2015), demonstrating the complexity in the microbial foundations of the lake food web. These extensive surveys also documented the occurrence of novel bacterial/archaeal phylotypes previously known to only occur in marine environments (Clingenpeel et al., 2011, 2013; Kan et al., 2011). However, the spatial distribution patterns of these microorganisms in the lake and the potential environmental drivers have not been fully addressed.

In this current study, we examined the size-fraction filtered (0.1, 0.8, and 3.0 μm nominal filtration) water samples from different locations of Yellowstone Lake, including surface water (3 and 10 m, photic zone), vent fluids, and mixing zones where vent waters mix with cold lake water. Both bacterial and archaeal community structures were assessed using 454 pyrosequencing of V1 andV2 regions of 16S ribosomal RNA genes. Efforts summarized herein focused on the free-living fractions (<0.8 μm) of microbial communities, with additional characterizations of microbial communities at bigger size fractions in two 10 m-depth, photic zone water samples. Geochemical profiling provided environmental context to the microbial community data, and relationships between microbial community structure and environmental variables were tested by using multivariate statistics. This study represents the first investigation of the spatial distribution of Bacteria and Archaea in a lake with documented geothermal inputs.

## Materials and Methods

### Sampling Locations and Geochemical Analyses

Surface photic zone, mixing zone, and vent water samples were collected with an ROV from lake regions and sites identified in previous USGS surveys (e.g., Morgan et al., 1977, 2007). Specifically, the sampling sites were: West Thumb Basin, Inflated Plain, Elliot’s Crater, and Southeast Arm at Yellowstone Lake, YNP (**Figure 1**). Detailed descriptions of sampling sites, locations, and depths were listed in **Table 1**. Geochemical analyses of water samples were as previously described (Lovalvo et al., 2010; Clingenpeel et al., 2011; Inskeep et al., 2015). To investigate mixing effects from vents on overlying water columns, two vertical sampling profiles of chemistry were conducted at Inflated Plain and Southeast Arm, respectively. In contrast to Inflated Plain, Southeast Arm is outside of the caldera and approximately 20 km from any major lake floor vent fields, and therefore experiences little impact from the lake hydrothermal activities.

**Table 1:** Description of sampling sites at Yellowstone Lake.

Sample^∗^	Location	Depth (m)	Total No. of sequences		Bac/Arc Ratio^#^
			Bacteria	Archaea	
SRF-YL340	West thumb basin(N44°24.976′ W110°31.505′)	2.5	22808	14527	4037.1
SRF-YL354	Southeast arm(N44°22.380′ W110°16.136′)	3	21651	12375	1652.8
SRF-YL347			23121	14335	3523.2
SRF-YL347(0.8–3.0 μm)	Inflated plain(N44°32.135′ W110°21.247)	10	24243	13157	-
SRF-YL347 (3–20 μm)			23020	12524	-
SRF-YL355			16267	13831	1838.0
SRF-YL355 (0.8–3.0 μm)	Southeast arm(N44°22.380’ W110°16.136′)	10	21177	14506	-
SRF-YL355 (3–20 μm)			24321	1616	-
MIX-YL360	Inflated plain(N44°32.085′ W110°21.260′)	33.3	26111	14444	535.2
MIX-YL370	West thumb basin(N44°24.976′ W110°31.505′)	52.1	18478	13854	3336.8
VNT-YL352	Elliot’s crater(N44°52.769′ W110°32.504′)	14.1	21115	12064	25.4
VNT-YL359	Inflated plain(N44°32.085′ W110°21.260′)	33.6	39795	12625	29.1
VNT-YL369	West thumb basin(N44°24.976′ W110°31.505′)	52.3	16074	13439	292.6

*^∗^Samples were analyzed on 0.1 μm fraction unless specified; SRF-, Surface; MIX-, Mixing; VNT-, Vent. ^#^Ratio of Bacteria to Archaea based on gene copy numbers estimated by qPCR (Einen et al., 2008); -, data not available.*

### Water Sampling and Epifluorescence Microscopy

Remotely operated vehicle operation and microbial and geochemical sampling methods were as previously described (Lovalvo et al., 2010; Clingenpeel et al., 2011; Kan et al., 2011). For microbial biomass collection, 100–300 L of lake/vent water was pumped through a 20 μm prefilter into 50 L carboys on the boat deck. Subsamples (10 mL) of filtrates passing through 20 μm were fixed in 1% glutaraldehyde for epifluorescence microscopy (Chen et al., 2001; Kan et al., 2006). The carboys were sterilized by either autoclaving or by soaking with 10% bleach followed by rinsing with autoclaved deionized water prior to each use. Following the protocol described for the Global Ocean Sampling (GOS; Rusch et al., 2007), surface and vent water was size fractionated by serial filtration through 3.0, 0.8, and 0.1 μm membrane filters. Each filter was sealed in a sterile plastic bag and frozen at -20°C for transport to the laboratory, where they were stored at -80°C until DNA extraction.

### DNA Extraction, Pyrosequencing, and Sequence Analyses

DNA extraction, 454 high-throughput pyrosequencing, and sequence analysis followed protocols we described previously (Clingenpeel et al., 2011; Kan et al., 2011). Briefly, genomic DNA was extracted from a quarter filter for each sample by using a phenol-chloroform extraction procedure. Hexadecyltrimethyl ammonium bromide (CTAB; 1% w/v) and sodium chloride (0.14 M) were used to remove polysaccharides and residual proteins during the DNA extraction. The V1 and V2 regions of the bacterial and archaeal 16S rRNA gene were amplified with barcoded primers (Bacteria: 27F and 533R; Archaea: A2Fa with A571R; Baker et al., 2003). The PCR products were pooled at equimolar concentrations according to their relative amplicon abundance and pyrosequenced using the 454 GS FLX Titanium sequencer of 454 Life Sciences (Branford, CT; Margulies et al., 2005) at the J.C. Venter Institute sequencing center. Pyrosequencing reads for both Bacteria and Archaea were screened and clustered using the UPARSE pipeline (Edgar, 2013) following the recommendations on the UPARSE website (http://www.drive5.com/usearch/manual/uparse_pipeline.html). Briefly, once barcodes and primer sequences were removed, all reads were trimmed to a length of 300 bp. Based on the Q scores reads with an average expected error greater than 1 were removed. For each sample the reads were subsampled to 16,074 bacterial reads and 12,064 archaeal reads. The sequences were clustered into operational taxonomic units (OTUs) based on 97% sequence similarity threshold. Chimeras were removed as an integral part of the UPARSE clustering method. All pyroread sequences are available in the GenBank SRA database under accession numbers SRX033214, SRX033251and SRX033252.

### Real Time PCR (QPCR)

Relative abundance of planktonic total Bacteria and Archaea were analyzed by Real-time qPCR, using the SYBR Green PCR kit (Qiagen) on an MJ research (Bio-Rad) qPCR machine by following previously described protocol (Einen et al., 2008). Briefly, bacterial and archaeal 16S rRNA genes were estimated by using primers 338f-518r and 931f-m1100r, respectively. A triplicate 10-fold dilution series of genomic DNA from *Shewanella oneidensis* MR-1 and *Halobacterium* sp. were used to generate standard curves for the Bacteria and Archaea, with biomass calculated based on estimated rRNA copy numbers for Bacteria (3.9) and Archaea (1.8) as described in Einen et al. (2008).

### Estimated Richness, Diversity Indices, and Multivariate Statistics

The richness estimator Chao1 was applied to estimate the number of missing species based on numbers of singletons and doubletons (Chao and Shen, 2010). Alpha Diversity (Shannon and Simpson indices) and beta diversity (Morisita-Horn index) were calculated using the program SPADE (Species Prediction and Diversity Estimation, Chao and Shen, 2010). The Bacteria and Archaea components of the community structure were separately analyzed by Non-metric Multidimensional Scaling (NMDS), employing the MDS procedure in SAS/STAT Software (v 9.3; SAS Institute Inc., Cary, NC, USA). Input to NMDS was a normalized data distance (or dissimilarity) matrix using the Bray–Curtis percent dissimilarity values based on sample relative abundances. For ease of interpretation, only the first two dimensions of a NMDS analysis were examined. In most cases, the scree plot (badness-of-fit criterion or stress plotted against number of dimensions) suggested that the first two dimensions were sufficient in defining the overall dimensionality of the input data matrix. Sample groupings suggested from NMDS results were assessed via Multi-Response Permutation Procedures (MRPP; PC-ORD software v 4, MjM Software Design, Gleneden Beach, OR, USA) for statistically significant grouping at *p* ≤ 0.05. Stress value less than 0.1 indicated a good ordination with little risk of misinterpretation of the distribution pattern (Clarke, 1993).

Variability in the original data explained by an NMDS dimension was assessed using an *R*^2^ value from a regression of the individual dimension scores vs. the original data matrix distance values. Assessing which specific taxonomic groups drove a particular NMDS result as well as examining the relationships between ancillary chemistry data and a given NMDS result was accomplished by correlating (Spearman rank) taxa relative abundances or environmental data values against the NMDS dimension scores. Significant (*p* ≤ 0.05 or 0.01) correlations indicate which bacterial/archaeal groups or environmental variables are driving differences in microbial community structure.

## Results

### Environmental Variables

**FIGURE 2:**
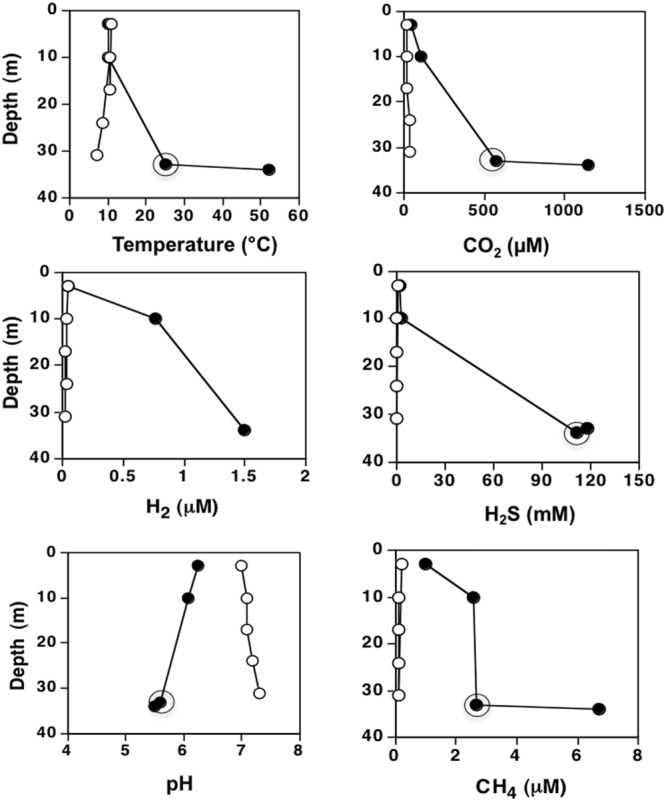
**Impacts of lake floor vents on overlying water column.** Profiles of selected variables from an Inflated Plain vent water column (filled symbols) vs. a Southeast Arm water column (open symbols) that was not vent influenced. Circles denoted the vent-lake water-mixing zone (not available for H_2_).

Metadata for the geochemistry measurements were summarized in **Table 2**. Analytes with more than three values missing or below detection limit including S_2_O_3_^2-^, PO_4_^3-^ and trace elements (Al, Mn, Fe, Ga, Se, Mo, Sb, Pb, V etc.) were not included in any summary or subsequent analyses. Waters from the deep mixing zone (MIX-YL360, 370) and thermal vents (VNT-YL352, 359, and 369) were mildly acidic, ranging from pH 5.6 to 6.6, compared to surface/shallow water from Southeast Arm (SRF-YL354 and 355, pH 7.0-7.1 with no vent impacts). Vent waters represented hydrothermal conditions of high temperature, high concentrations of gasses and low O_2_. Temperature for surface waters ranged from 11.2 to 13°C, while vent waters had pronounced higher temperatures (48–65.5°C). A generally increasing trend from surface to vent waters was also observed for NH_4_^+^, CH_4_, CO_2_, H_2_S, SO_4_^2-^, and DIC (dissolved inorganic carbon), while O_2_ decreased from 313 to 118 μM. All other environmental measurements such as DOC (dissolved organic carbon), anions, cations, and trace elements were generally similar and no conspicuous trend was found (**Table 2**). Aqueous geochemistry, gasses and temperature were examined within the water column atop a particularly active vent in the Inflated Plain (within the caldera) and compared against a water column not associated with any known vent(s) in the Southeast Arm (out of the caldera; **Figure 1**). Interest was primarily on the vent water, the mixing zone (defined as that location directly above the vent where water temperature decreased below the vent water), and then at depths of 3 and 10 m (**Figure 2**). Because of the high vent output, no obvious mixing zone of H_2_ was observed in the lake (**Figure 2**), although clearly H_2_ was locally enriched in vent emissions and the water column overlying highly active vents or mixing zone samples containing vent fluids (**Table 2**, **Figure 2**).

**Table 2:** Major geochemistry measurements for the collected samples at Yellowstone Lake.

	Variable	SRF-YL340	SRF-YL354	SRF-YL347^∗^	SRF-YL355	MIX-YL360	MIX-YL370	VNT-YL352	VNT-YL359	VNT-YL369
	pH	6.8	7.0	6.1	7.1	5.6	6.6	6.4	5.6	6.0
	Temp (°C)	11.2	13.0	12.2	12.3	25.5	26.0	65.5	48.0	56.5
	DOC (μM)	166	185	166	343	299	-	148	390	-
Anions	Cl (mM)	0.14	0.13	0.14	0.13	0.15	0.17	0.39	0.14	1.09
	F (μM)	31.5	29.0	33.0	28.0	33.0	40.0	35.0	32.0	147.0
	NO_3_ (μM)	1.30	0.40	0.07	0.05	0.58	1.80	0.59	0.53	4.82
	SO_4_ (mM)	0.07	0.08	0.08	0.07	0.08	0.09	0.08	0.20	0.11
Cations	Ca (mM)	0.13	0.15	0.17	0.15	0.15	0.16	0.18	0.14	0.15
	K (mM)	0.04	0.04	0.06	0.04	0.04	0.04	0.07	0.04	0.11
	Mg (μM)	95.1	85.6	131.0	85.1	80.6	85.1	97.2	80.1	72.4
	Na (mM)	0.39	0.40	0.39	0.40	0.43	0.53	0.89	0.42	2.53
	NH_4_ (μM)	2.8	3.9	2.6	6.2	37.5	27.2	45.0	8.2	23.4
	Si (mM)	0.16	0.15	0.16	0.15	0.14	0.15	0.32	0.13	0.81
Dissolved gasses	CH_4_ (μM)	0.1	0.2	2.6	0.1	2.7	-	2.3	6.1	7.6
	CO_2_ (mM)	0.02	0.01	0.10	0.02	0.57	-	0.48	1.15	2.36
	DIC (mM)	0.56	0.60	0.60	1.80	0.80	-	1.30	1.30	5.60
	H_2_ (nM)	3	47	762	33	386	-	660	1499	32
	O_2_ (μM)	313.0	237.5	261.0	273.1	23.1	211.0	118.8	-	197.4
	DS (μM)	-	1.0	2.5	0.1	118.1	0.3	21.7	97.6	11.0
Trace elements	As (μM)	0.26	0.18	0.19	0.18	0.18	0.26	0.40	0.17	3.98
	B (μM)	10.74	8.76	9.19	9.08	10.90	12.42	27.49	10.80	-
	Ba (μM)	0.17	0.22	0.26	0.06	0.81	0.46	0.26	0.34	0.11
	Cs (μM)	0.26	0.01	0.01	0.01	0.01	0.01	0.04	0.01	0.43
	Li (μM)	-	6.83	6.93	6.78	7.09	8.82	18.14	7.01	39.88
	Rb (μM)	0.07	0.03	0.03	0.03	0.02	0.03	0.08	0.03	0.55
	Sr (μM)	0.52	0.56	0.56	0.56	0.57	0.58	0.92	0.56	0.55
	W (μM)	0.05	0.01	0.01	0.01	0.01	0.02	0.02	0.01	0.34
	Zn (μM)	0.13	0.14	0.07	0.15	0.09	0.36	-	-	0.13

*-, Missing data or below detection limit; DOC, dissolved organic carbon; DIC, dissolved inorganic carbon; DS, dissolved sulfide.*

*^∗^denotes a water column overlying a highly active vent.*

*Due to missing values or below detection, parameters including S_*2*_O_*3*_^*2*-^, PO_*4*_^*3*-^ and trace elements (Al, Mn, Fe, Ga, Se, Mo, Sb, Pb, V etc.) are not listed.*

### Microbial Community Composition

A total of 519,779 high-quality 16S rRNA gene sequences were obtained from the lake water samples. Of these, 329,878 came from the primers for Bacteria with an average 23,563 reads per sample and 189,901 for Archaea, averaging 14,483 reads per sample (except low yield for the Southeast Arm photic sample SRF-YL355 3 μm fraction, **Table 1**). Quantitative PCR results indicated that archaeal sequences reflected minority abundance relative to Bacteria, and Bacteria/Archaea ratio varied from ∼4,037 (surface water) to ∼25 (vent water; **Table 1**).

**FIGURE 3:**
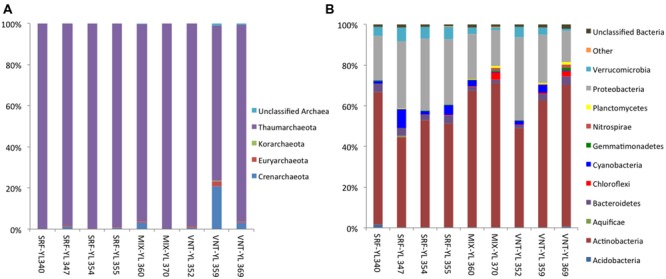
**Composition of free-living archaeal **(A)** and bacterial communities **(B)** in Yellowstone Lake waters (phylum level)**.

In all samples the Thaumarchaeota was the most dominant archaeal group in the lake (**Figure 3A**). The Crenarchaeota made up a significant portion (20.7%) of the reads from the Inflated Plain vent and were also seen at low levels (<4%) in the waters above that vent. Low levels of Crenarchaeota were also seen in other vent waters (0.7–3.3%). The Euryarchaeota were only found at significant levels in the West Thumb cone vent at 8.5% of that sample’s reads and at 2.5% of the reads from an Inflated Plain vent. In all other samples the Euryarchaeota made up less than 1% of the archaeal reads. For the Bacteria, Actinobacteria (freshwater acI and acIV), Bacteroidetes (Chitinophagaceae), Cyanobacteria (*Prochlorococcus*-like), Alphaproteobacteria (*Pelagibacter*/SAR11-like), Betaproteobacteria (Burkholderiales *and* Methylophilales), and Verrucomicrobia (Puniceicoccaceae) were the major groups in the lake (**Figure 3B**). Although microbial diversity *per se* was not the focus for the current study, the results agreed well with previous characterization of population structures for Bacteria, where detailed phylogeny was conducted based on comparison of high-quality 16S rRNA gene sequences with full-length 16S rRNA gene clones (Clingenpeel et al., 2011). Freshwater Actinobacteria was the most dominant group in the lake ranging from 44.3% (SRF-YL347) to 70.8% (MIX-YL370) of the total reads. Proteobacteria (mainly Alpha and Beta) were the second most abundant group in all the sampled waters, with abundances varying from 15.5% (VNT-YL369) to 40.8% (VNT-YL352). The most abundant Cyanobacteria were found in the 10 m depth photic zone samples at 4.9% (SRF-YL355) and 9.2% (SRF-YL347) of the reads. Finally, two other groups were present at significant levels in all water samples: Bacteroidetes at an average abundance of 2.7% and Verrucomicrobia at an average abundance of 3.8% of the total reads. The sequences obtained for both the Bacteria and Archaea provided considerable resolution for identifying important phylotypes present in the water samples and detailed taxonomic classifications were listed in **Supplementary Tables S1** and **S2**

### Community Composition In Different Size Fractionations

**FIGURE 4:**
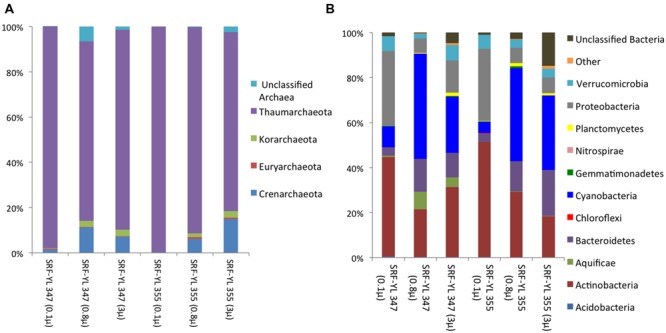
**Phylum level composition of archeal **(A)** and bacterial **(B)** communities in samples SRF-YL347 and SRF-YL355 with different size fractionations: 0.1 μ (0.1–0.8 μm), 0.8 μ (0.8–3.0 μm), and 3 μ (3.0–20 μm)**.

In addition to 0.1–0.8 μm filter classification, microbial communities in the 0.8–3.0 and 3.0–20 μm filtration classes from stations SRF-YL347 and SRF-YL355 (both 10 m photic zone water) were also characterized (**Figure 4**). The Crenarchaeota and Korarchaeota were more abundant in bigger size fractionations (0.8–3.0 and 3.0–20 μm), whereas the smallest size fraction (0.1–0.8 μm) was almost entirely Thaumarchaeota (**Figure 4A**). Bacterial communities also showed size distribution patterns: free-living fractions contained more Acidobacteria, Actinobacteira, Alphaproteobacteria, and Betaproteobacteria, while the Aquificae, Bacteroidetes, Cyanobacteria, Deltaproteobacteria, and Planctomycetes were more abundant in the bigger size fractions (**Figure 4B**). Estimated OTU richness (at 97%) and diversity indices showed larger size fractions contained higher diversity for both Archaea and Bacteria (**Table 3**). The beta diversity measure (Morisita-Horn index) confirmed that free-living Archaea and Bacteria were distinct from the bigger size fractions.

**Table 3:** Coverage-based estimated OTU richness (at 97%) and diversity indices for Archaea and Bacteria in Yellowstone Lake.

Sample^∗^	Archaea			Bacteria		
	Chao1	*H* (Shannon index)	*D* (Simpson index)	Chao1	*H* (Shannon index)	*D* (Simpson index)
SRF-YL340	4.0 (0.4)	0.272 (0.006)	0.859 (0.640)	234.6 (20.5)	2.796 (0.007)	0.101 (0.023)
SRF-YL354	6.0 (0.0)	0.384 (0.008)	0.821 (0.589)	288.3 (39.7)	2.746 (0.008)	0.113 (0.027)
SRF-YL347	19.0 (1.9)	0.531 (0.01)	0.782 (0.586)	221.1 (20.0)	2.819 (0.008)	0.104 (0.025)
SRF-YL347 (0.8–3.0 μm)	33.3 (3.4)	1.594 (0.012)	0.374 (0.124)	305.1 (44.2)	2.456 (0.008)	0.185 (0.053)
SRF-YL347 (3–20 μm)	31.5 (2.3)	1.118 (0.014)	0.574 (0.300)	338.5 (19.9)	3.046 (0.009)	0.116 (0.028)
SRF-YL355	14.0 (4.5)	0.432 (0.009)	0.807 (0.622)	260.0 (38.7)	2.826 (0.009)	0.109 (0.025)
SRF-YL355 (0.8–3.0 μm)	43.1 (3.7)	1.153 (0.013)	0.554 (0.286)	254.0 (25.2)	2.72 (0.01)	0.163 (0.040)
SRF-YL355 (3–20 μm)	18.0 (0.0)	1.629 (0.032)	0.310 (0.104)	326.0 (49.2)	3.029 (0.008)	0.096 (0.021)
MIX-YL360	24.1 (1.8)	0.847 (0.010)	0.613 (0.366)	299.3 (28.6)	2.646 (0.007)	0.119 (0.031)
MIX-YL370	21.5 (17.1)	0.615 (0.007)	0.673 (0.449)	342.1 (56.0)	2.745 (0.009)	0.114 (0.028)
VNT-YL352	21.3 (3.4)	0.681 (0.009)	0.665 (0.432)	245.0 (28.8)	2.575 (0.008)	0.138 (0.036)
VNT-YL359	47.0 (6.5)	1.626 (0.012)	0.353 (0.125)	259 (18.1)	2.769 (0.006)	0.103 (0.025)
VNT-YL369	46.0 (23.6)	0.993 (0.01)	0.535 (0.295)	249.8 (21.8)	2.829 (0.009)	0.110 (0.025)

*^∗^Samples were analyzed on 0.1 μm fraction unless specified.*

*Standard errors for the estimates were shown in parentheses.*

**FIGURE 5:**
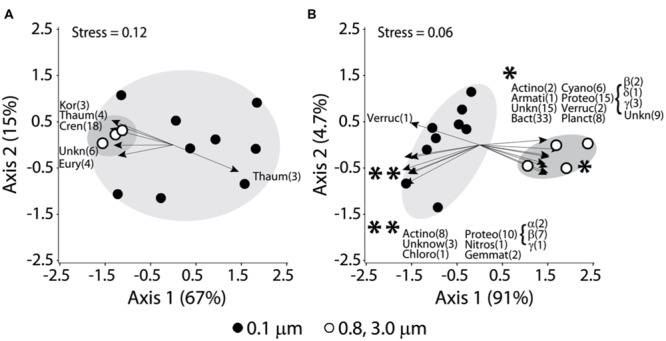
**Non-metric multidimensional scaling (NMDS) plots for microbial distributions with different size fractionations and major phyla (OTU numbers) responsible for the distribution patterns based on significant Spearman correlations (as shown by arrows): **(A)** Archaea; **(B)** Bacteria.** Open symbols were 0.8–3.0 and 3.0–20 μm, while filled symbols were free-living fraction (0.1–0.8 μm). Abbreviations for Archaea: Cren, Crenoarchaeota; Eury, Euryarchaeota; Kor, Korarchaeota; Thaum, Thaumarchaeota; Unkn, unknown/unclassified. Abbreviations for Bacteria: Acido, Acidobacteria; Actino, Actinobacteria; Armati, Armatimonadetes (OP10), Bact, Bacteroidetes; Cyano, Cyanobacteria; Chloro, Chloroflexi; Gemmat, Gemmatimonadetes; Nitros, Nitrospira; Planct, Planctomycetes; Proteo, Proteobacteria (α, β, γ, δ-subdivision); Verruc, Verrucomicrobia; Unkn, unknown/unclassified. Only phyla significantly correlated with the scores for a NMDS axis (Archaea, *p* < 0.05; Bacteria, *p* < 0.01) were shown. MRPP analyses showed significantly distinct groupings (shadowed) for (**A**; *p* = 0.016) and (**B**; *p* = 0.00014). Arrow length represented the strength of significant correlations. The asterisks (^∗^ and ^∗∗^) in **(B)** were for ease of labeling and showed the phyla associated with the respective group of arrows.

The NMDS-based community composition comparisons, examining relative abundances at the genus level (97% cutoff), also demonstrated differences between archaeal and bacterial communities (**Figures 5A,B**). Although not as distinct as Bacteria, the archaeal communities (all OTUs) formed groups between the different fractions (**Figure 5A**, MRPP *p* = 0.016). Diverse groups of Archaea were prominent in the bigger size fractions (0.8–3.0 and 3.0–20 μm) such as Crenarchaeota (Thermoprotei), Korarchaeota (Koararchaeum), Euryarchaeota (Methanomicrobia etc.), Thaumarchaeota (Nitrosopumilales) and unknown archaeal groups, while only Thaumarchaeota (different OTUs from Nitrosopumilales) was significantly related to free-living Archaea (**Figure 5A**). In contrast, smaller bacterial size fraction (0.1–0.8 μm) grouped quite distinctly from the bigger sized components (0.8–3.0 and 3.0–20 μm; **Figure 5B**). Both stress values and MRPP results suggested that the differences between the sizes were statistically significant.

The appearance and relative abundance of certain groups of Bacteria accounted for the difference between different size fractions (**Figure 5B**). Actinobacteria (Micrococcineae, Acidimicrobineae, Mycobacterium, Ilumatobacter), Chloroflexi, Gemmatimonadetes (Gemmatimonas), Nitrospira, Verrumicrobia, and unknown Bacteria were of greater influence on smaller size fraction communities. In contrast, the Actinobacteria (Actinomycetales, Acidimicrobineae), Armatimonadetes (Armatimonas), Bacteroidetes (33 OTUs), Cyanobacteria (6 OTUs), Planctomycetes (8 OTUs), Verrucomicrobia and unknown Bacteria dominated the larger-sized fractions (**Figure 5B**).

**FIGURE 6:**
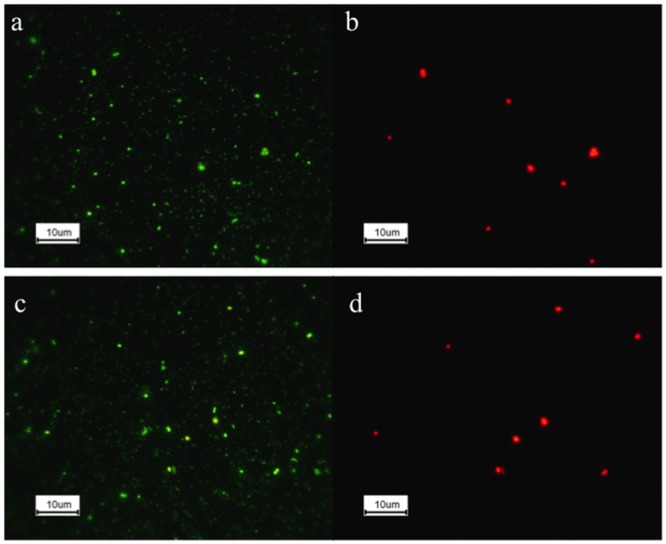
**Epifluorescence microscopic images for Yellowstone Lake waters SRF-YL347 **(a,b)** and SRF-YL355 **(c,d)**. (a,c)**, total microbial communities; **(b,d)**, same field as **(a,c)** but with blue light excitation to show photosynthetic cells.

Our epifluorescence microscopic observation confirmed the photosynthetic cells including Cyanobacteria were bigger than most non-pigmented cells (**Figures 6a** vs. **6b, 6c** vs. **6d**). As the biggest bacterial phylum, Proteobacteria accounted for the distribution of both free-living and bigger size fractions, however, distinct subgroups and OTUs were identified as significant components: Alpha, Beta and Gammaproteobacteria for free-living while Beta, Delta, Gammaproteobacteria and unknown Proteobacteria for bigger size fractionations (**Figure 5B**).

### Spatial Distribution of Free-Living Microbial Communities and Their Relationship with Environmental Parameters

**FIGURE 7:**
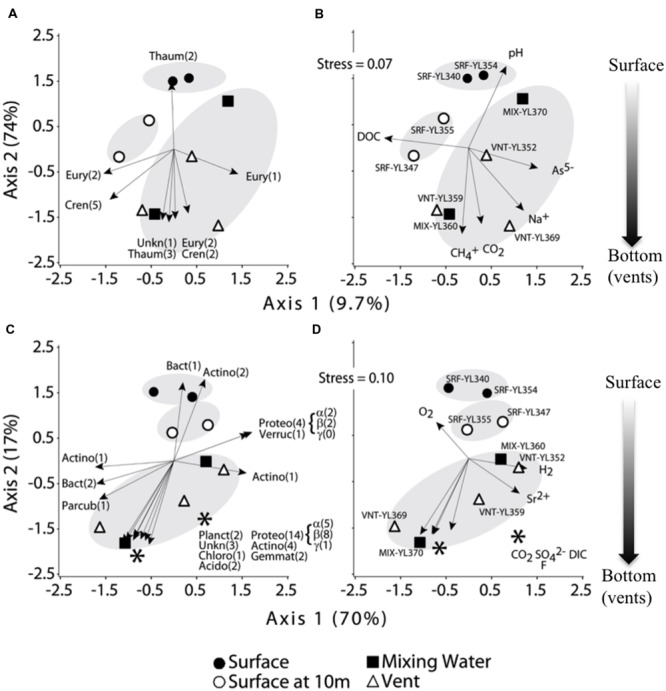
**Distribution pattern (NMDS plots) of free-living Archaea **(A,B)** and Bacteria **(C,D)**.** Major phyla responsible for the distribution patterns of Archaea and Bacteria were shown in **(A)** and **(C)**, and correspondence of environmental variations were shown in **(B)** and **(D)**. Abbreviations for Archaea: Cren, Crenarchaeota; Eury, Euryarchaeota; Thaum, Thaumarchaeota; Unkn, unknown/unclassified. Abbreviations for Bacteria: Acido, Acidobacteria; Actino, Actinobacteria; Bact, Bacteroidetes; Chloro, Chloroflexi; Gemmat, Gemmatimonadetes; Parcub, Parcubacteria (OD1); Proteo, Proteobacteria (α, β, γ-subdivision); Verruc, Verrucomicrobia; Unkn, unknown/unclassified. Only phyla significantly correlated with the scores for a NMDS axis (Archaea, *p* < 0.05; Bacteria, *p* < 0.01) were shown. MRPP analyses showed significantly distinct groupings (shadowed) for (**A**; *p* = 0.016) and (**B**; *p* = 0.00014). Arrow length represented the strength of significant correlations. The asterisks (^∗^ and ^∗∗^) in panel **(C)** and **(D)** were for ease of labeling and showed the phyla associated with the respective group of arrows.

Further analyses of microbial distributions focused on free-living microbes only. NMDS analyses demonstrated that both Archaea and Bacteria exhibited a distribution pattern from 3 m depth (SRF-YL340, 354) to vent waters (VNT-YL352, 359, 369), with 10 m depth (SRF-YL347, 355) and mixing zone waters (MIX-YL360, 370) in between (**Figure 7**). Bacterial populations from surface waters and vent communities were grouped separately, but the mixing water did not form a distinct group. Instead, both archaeal and bacterial communities from mixing zones were more similar to the vent waters at the same locations (VNT-YL359 to MIX-YL360; VNT-YL369 to MIX-370; **Figures 7B,D**). Estimated richness and diversity measures (alpha and beta diversity indices) for free-living microbes indicated that vent waters contained higher diversity of Archaea than surface and mixing water samples. In contrast, bacterial community structures shared higher similarity than Archaea among all the water samples and no clear increasing or decreasing trend was observed with depth (**Table 3**).

Microbial groups responsible for the distribution patterns were shown in **Figure 7A** (Archaea) and **Figure 7C** (Bacteria). Thaumarchaeota (Nitrosopumilales) correlated to surface water archaeal distribution while Euryarchaeota (Methanomicrobia and unknown Euryarchaeota), Crenarchaeota (Thermoprotei, Desulfurococcales, Ignisphaera, and Pyrobaculum), Thaumarchaeota (different OTUs from Nitrosopumilales), and unknown Archaea were mainly responsible for the vent archaeal communities (**Figure 7A**). As Archaea, Bacteria showed similar distribution patterns: Bacteroidetes (Sediminibacterium) and Actinobacteria (Cryobacterium and Micrococcineae) were positively related to the surface bacterial community, and a diverse group of *Bacteria* responded to depth profiles, such as Bacteroidetes (Arcicella and Sediminibacterium), Parcubacteria (unknown), Planctomycetes (Phycisphaera and Planctomycetaceae), Proteobacteria (Alpha, Beta and Gammaproteobacteria), Actinobacteria (Mycobacterium, Micrococcineae, and Ilumatobacter), Chloroflexi (Anaerolineaceae), Acidobacteria (Gp6), Gemmatimonadetes (Gemmatimonas), and unknown Bacteria (**Figure 7C**).

Correlation analyses revealed that the distribution of the free-living microbial communities correlated well with water geochemistry measured in this study (**Figures 7B,D**). Surface water (photic zone) archaeal communities were associated with increasing pH and DOC, while the deep vent water communities were associated with hydrogeothermal features including increasing concentrations of CH_4_, CO_2_, Na^+^ and As^5-^ (**Figure 7B**). Dissolved O_2_ was identified as the significant driver for surface water free-living bacterial community, which was distinguished from deep mixing zone/vent waters by increasing concentrations of CO_2_, H_2_, SO_4_^2^, F^-^, DIC and Sr^2+^ (**Figure 7D**).

## Discussion

This study followed the sampling protocol in the GOS expedition (Rusch et al., 2007) of three size fractionations: 0.1–0.8, 0.8–3.0, and 3.0–20 μm. Therefore, we had the opportunity to investigate microbial communities from different size fractions. Microscopic observations and cell enumeration (this study and Clingenpeel et al., 2011) have suggested that the 0.1–0.8 μm fraction represented the free-living microorganisms, while fractions >3.0 μm were mostly particle associated. Our results (this study and Clingenpeel et al., 2011) confirmed that cell counts for particle-attached microbes were generally <20–50% of the total cell counts (Kirchman and Mitchell, 1982; Kirchman, 1983; Pernthaler et al., 1996). Larger-sized fractions that would likely include particle-associated microbes (>3 μm) contain larger and metabolically more active microbial groups (Stevenson, 1978; Gasol et al., 1995; Pernthaler et al., 1996). For instance, Cyanobacteria have been found to dominate in particle-associated fraction due to their size (Rosel et al., 2012), which is consistent with the epifluorescence microscopy images obtained in the current study (**Figure 6**). Further, 33 OTUs of Bacteroidetes were identified associated with the bigger size fractions (**Figure 5B**). These microbes have been considered crucial degraders of high molecular weight organic matter (Kirchman, 2002; Thomas et al., 2011). Another important group occurring more commonly within the bigger size fractionations was Planctomycetes, which has been found widely distributed in both freshwaters and marine environments (reviewed by Lage and Bondoso, 2012 and references therein). Due to their well-known distinctive morphology with cells forming rosettes connected by a non-cellular stalk (Fuerst, 1995), these microorganisms were easily collected by bigger pore sized filters. The higher estimated richness and diversity for both Archaea and Bacteria (**Table 3**), indicates greater richness and diversity is associated with organic particles in the water column or in multispecies aggregates as observed by Rosel et al. (2012) and Crespo et al. (2013). Due to the size fractionation approach applied, with emphasis on free-living organisms, this lake’s true microbial diversity/activities is likely underestimated, and represents an intriguing target for future studies.

In big lakes such as Yellowstone Lake, environmental gradients influence the distribution of aquatic microorganisms (**Figure 7**). Previous studies demonstrated that oxygen gradients were associated with bacterial distributions (Cole et al., 1993; Lehours et al., 2007; Salcher et al., 2008, 2010), and similar patterns were observed in this study (**Figure 7D**), although the samples obtained in the current study did not derive from a stratified lake and O_2_ gradients almost certainly resulted from the lake bottom vents. In addition, lake microbial communities have also been shown to respond to other environmental parameters including nutrients (Cole et al., 1993; Casamayor et al., 2000) and salinity (Baricz et al., 2014). Hydrothermal vents on the bottom of Yellowstone Lake significantly contribute to highly localized temperature, pH and geochemistry profiles (**Table 2** and Inskeep et al., 2015), and not unexpectedly, these complicated gradients shaped the distribution of microbial populations (**Figures 7B,D**). For instance, apparent thermophilic Archaea (e.g., Crenarchaeota-Thermoprotei) were found throughout the lake, but were highly enriched in mixing zone and vent waters (**Figure 7A**, **Supplementary Tables S1** and **S2**), suggesting the significant geothermal impacts on these specific lake environments. As noted from **Figure 7B**, pH was one of the significant factors controlling the distribution of archaeal communities in the lake. While not significant at *p* = 0.05, temperature was correlated with both NMD axes for Archaea and Bacteria (data not shown). Consistent with this observation, both pH and temperature were identified as controlling factors in selecting microorganisms and determining their distributions from a review of 15 typical freshwater microbial groups found in 15 diverse European lakes (Lindstrom et al., 2005).

Previous reports have shown that the availability of trace elements, major nutrients and the distribution of major energy sources influenced the diversity and productivity of biological communities in Yellowstone Lake (Lovalvo et al., 2010; Clingenpeel et al., 2011, 2013; Kan et al., 2011; Yang et al., 2011; Inskeep et al., 2015). In the current study, multivariate statistics corroborate that hydrothermal vent-associated geochemistry (e.g., CH_4_, CO_2_, pH, DOC, Na^+^, As^5-^, and O_2_, H_2_, CO_2_, SO_4_^2-^, DIC, F^-^, Sr^2+^) were the major drivers for Archaea and Bacteria diversity, and their distributions, respectively (**Figures 7B,D**). In comparison to measurements of general geothermal features (e.g., Langner et al., 2001; Macur et al., 2004; D’Imperio et al., 2008), emissions of hydrothermal vent gasses including CO_2_, and CH_4_ from the lake vents were considerably higher than terrestrial hot springs in the Yellowstone Park (**Table 2**; Inskeep et al., 2015). H_2_ level was significant throughout the lake, and clearly it was locally enriched in vent emissions (**Table 2**; Clingenpeel et al., 2011). Since the H_2_ growth threshold concentrations were either equivalent to or below nM levels (Conrad et al., 1983), we hypothesized that H_2_ throughout the lake served as significant energy sources for broad microbial populations and activities, as noted from Yellowstone’s hot springs (Spear et al., 2005; D’Imperio et al., 2008). As a result, methanogens (e.g., Methanomicrobia) were more commonly found in hydrothermal vents and their distribution patterns indicated close relationship to CH_4_ and CO_2_ concentrations (**Figures 7A,B**, **Supplementary Tables S1** and **S2**). Further, putative nitrifying Archaea (Nitrosopumilales) were widely distributed in the lake demonstrating their potential association to nitrifier-relevant concentrations of carbon (CO_2_). High temperature, H_2_S, SO_4_^2-^, H_2_, and low O_2_ in vent waters favored growth of thermophilic Crenarchaeota of the orders Desulfurococcales and Thermoproteales, in which numerous cultivated representatives have been shown to be capable of gaining energy via anaerobically respiring sulfur (Huber and Stetter, 2006; Macur et al., 2013). Our results showed that both Desulfurococcales and Thermoproteales were more enriched in deep vents VNT-YL359 (2.1 and 7.6%) and VNT-YL369 (2.1 and 0.2%).

Similar distribution patterns of free-living bacterial populations in the lake were also observed (**Figures 7C,D**). The population structures positively correlated with the relevant geochemical signatures such as H_2_, CO_2_, and SO_4_^2-^. Substantial CH_4_ and CO_2_ levels translated to significant reduced one-carbon compound metabolisms as evidenced by presence of methylotrophs (*Methylocystis*, and *Methylotenera*; **Supplementary Tables S1** and **S2**), which are commonly found in marine and freshwater environments (reviewed by Anthony, 1982; Hanson and Hanson, 1996; Chistoserdova et al., 2009). In addition, higher concentrations of H_2_S, and SO_4_^2-^- along the oxygen gradient would potentially provide habitats suitable for sulfur-oxidizing and sulfate-reducing Bacteria. Recent efforts to characterize the microbial communities from several vent sites in Yellowstone Lake indicated that sulfur-oxidizing bacteria were important in sulfidic habitats (Yang et al., 2011), and our data documented the presence of similar sulfur-oxidizing groups including Aquificales (*Sulfurihydrogenibium*) and Proteobacteria (*Thiovirga, Thiobacillus, Thiothrix*, and *Sulfuricurvum*; **Supplementary Tables S1** and **S2**). Further, although not abundant among the total community, sulfate-reducing Deltaproteobacteria (Desulfobacteraceae) were also present in vent and deep waters. Recent metagenomic and functional gene characterizations of vent waters and streamers in Yellow Stone Lake have further supported the geochemical controls (elevated CO_2_, S, H_2_, and CH_4_ etc.) on microbial community structures in the deep thermal ecosystems (Inskeep et al., 2015). Although certain functional groups/pathways identified were more favorable and dominant in vents, we realized that physiological inference was limited based on the high-throughput sequences because of: (1) constrained taxonomic resolution of short read lengths; and (2) limited current knowledge on known metabolic capabilities for Bacteria and Archaea in general. Nevertheless, our results indicate archaeal and bacterial distributions were strongly influenced by geothermal inputs of Yellowstone Lake.

Another possible explanation for the microbial distributions was mixing events, which would exert pronounced influence on water bodies especially those with longer water retention time. These mixing events would dilute emissions of hydrothermal vents with the overlying water column and thus impact the entire lake geochemistry and distribution of microorganisms. For instance, the free-living microorganisms from the mixing zone water (>30 m depth) tended to be more similar to the deep vents at the same locations (VNT-YL359 and MIX-YL360; VNT-YL369 and MIX-YL370), except the Archaea at stations VNT-YL369 and MIX-YL370 (**Figures 6b,d**). Clearly, vent emissions significantly influenced the mixing zone samples. We conclude that both archaeal and bacterial distributions were reliant on the vent-provided chemicals in the lake, but the microbial composition in deeper water was obviously overwhelmed by mixing events, which was clearly demonstrated by the water chemistry profiles in Inflated Plain (**Figure 2**). Wind-generated (and other mixing) currents would also disperse the geochemicals and microorganisms throughout the lake, as demonstrated by H_2_ profiles (**Figure 2** and **Table 2**). In addition to vent emission, other potential sources of lake H_2_ could derive from hydrothermal activity, nitrogenase activity or eukaryotic algae (Hanson and Hanson, 1996; Melis and Happe, 2001). Nevertheless, microbial distribution correlated with hydrothermal activities and the associated geochemistry (CH_4_, SO_4_^2-^, H_2_, CO_2_, O_2_, DOC, DIC, pH, and other trace metals), suggesting that these microorganisms were likely involved in cycling geochemicals associated with the vent emissions. Obviously, depending on the relative association with lake vents, the lake food chain is not simple. In addition to phototrophy (as noted the dominancy of Cyanobacteria), chemolithotrophy involving hydrogen, carbon, and sulfur metabolism are likely other major energy platforms in this lake.

## Author Contributions

JK, SC, and TM collected the data and wrote the manuscript; CD helped with the data analyses and multivariate statistics; RM and WI analyzed water and gas chemistry; KN led and oversaw the project.

## Conflict of Interest Statement

The authors declare that the research was conducted in the absence of any commercial or financial relationships that could be construed as a potential conflict of interest.

## Supplementary Material

TABLE S1 **Detailed identification of archaeal sequences and their classification at Phylum, Class, Order, Family, and Genus levels**.

TABLE S2 **Detailed identification of bacterial sequences and their classification at Phylum, Class, Order, Family, and Genus levels**.

## References

[B1] Anthony C. (1982). *The Biochemistry of Methylotrophs.* London: Academic Press.

[B2] Baker G. C. Smith J. J. Cowan D. A. (2003). Review and re-analysis of domain-specific 16S primers. *J. Microbiol. Methods* 55 541–555. 10.1016/j.mimet.2003.08.009 14607398

[B3] Balistrieri L. I. Shanks W. C. Cuhel R. L. Aguilar C. Klump J. V. (2007). “The influence of sublacustrine hydrothermal vent fluids on the geochemistry of yellowstone lake,” in *Integrated Geoscience Studies in the Greater Yellowstone Area – Volcanic, Tectonic, and Hydrothermal Processes in the Yellowstone Geoecosystem* ed. Morgan L. A. (Boulder, CO: U.S. Geological Survey Professional Paper 1717) 173–199.

[B4] Baricz A. Coman C. Andrei A. S. Muntean V. Keresztes Z. G. Pausan M. (2014). Spatial and temporal distribution of archaeal diversity in meromictic, hypersaline Ocnei Lake (Trasylvanian Basin, Romania). *Extremophiles* 18 399–413. 10.1007/s00792-013-0625-6 24414798

[B5] Benson N. G. (1961). *Limnology of Yellowstone Lake in Relation to the Cutthroat Trout* Research Report 56 (Washington DC: US Fish and Wildlife Service) 33.

[B6] Carlsson P. Caron D. (2001). Seasonal variation of phosphorus limitation of bacterial growth in a small lake. *Limnol. Oceanogr.* 46 108–120. 10.4319/lo.2001.46.1.0108

[B7] Casamayor E. Schafer H. Baneras L. Pedro’s-Alio C. Muyzer G. (2000). Identification of and spatio-temporal differences between microbial assemblages from two neighboring sulfurous lakes: comparison by microscopy and denaturing gradient gel electrophoresis. *Appl. Environ. Microbiol.* 66 499–508. 10.1128/AEM.66.2.499-508.2000 10653710 PMC91855

[B8] Chao A. Shen T. J. (2010). *Program SPADE (Species Prediction And Diversity Estimation). Program and User’s Guide.* Available at: http://chao.stat.nthu.edu.tw

[B9] Chen F. Lu J. R. Binder B. Hodson R. E. (2001). Enumeration of viruses in aquatic environments using SYBR gold stain: application of digital image analysis and flow cytometer. *Appl. Environ. Microbiol.* 67 539–545. 10.1128/AEM.67.2.539-545.2001 11157214 PMC92618

[B10] Chistoserdova L. Kalyuzhnaya M. G. Lidstrom M. E. (2009). The expanding world of methylotrophic metabolism. *Annu. Rev. Microbiol.* 63 477–499. 10.1146/annurev.micro.091208.073600 19514844 PMC2827926

[B11] Clarke K. R. (1993). Nonparametric multivariate analyses of changes in community structure. *Austra. J. Ecol.* 18 117–143. 10.1111/j.1442-9993.1993.tb00438.x

[B12] Clingenpeel S. Kan J. Macur R. E. Woyke T. Lovalvo D. Varley J. (2013). Yellowstone Lake Nanoarchaeota. *Front. Microbiol.* 4:274 10.3389/fmicb.2013.00274 PMC3769629 24062731

[B13] Clingenpeel S. Macur R. E. Kan J. Inskeep W. P. Lovalvo D. Varley J. (2011). Yellowstone Lake: high energy geochemistry and rich bacterial diversity. *Environ. Microbiol.* 13 2172–2185. 10.1111/j.1462-2920.2011.02466.x 21450005

[B14] Cole J. Pace M. Caraco N. Steinhart G. (1993). Bacterial biomass and cell size distributions in lakes: more and larger cells in anoxic waters. *Limnol. Oceanogr.* 38 1627–1632. 10.4319/lo.1993.38.8.1627

[B15] Conrad R. Aragno M. Seiler W. (1983). The inability of hydrogen bacteria to utilize atmospheric hydrogen is due to threshold and affinity for hydrogen. *FEMS Microbiol. Lett.* 18 207–210. 10.1111/j.1574-6968.1983.tb00479.x

[B16] Crespo B. G. Pommier T. Fernandez-Gomez B. Pedros-Alio C. (2013). Taxonomic composition of the particle-attached and free-living bacterial assemblages in the Northwest Mediterranean Sea analyzed by pyrosequencing of the 16S rRNA. *Microbiology Open* 2 541–552. 10.1002/mbo3.92 23723056 PMC3948605

[B17] D’Imperio S. Lehr C. R. Oduro H. Druschel G. Kühl M. McDermott T. R. (2008). Relative importance of H_2_ and H_2_S as energy sources for primary production in geothermal springs. *Appl. Environ. Microbiol.* 74 5802–5808. 10.1128/AEM.00852-08 18641166 PMC2547044

[B18] Edgar R. C. (2013). UPARSE: highly accurate OTU sequences from microbial amplicon reads. *Nat. Methods* 10 996–998. 10.1038/nmeth.2604 23955772

[B19] Einen J. Thorseth J. H. Ovreas L. (2008). Enumeration of Archaea and Bacteria in seafloor basalt using real-time quantitative PCR and fluorescence microscopy. *FEMS Microbiol. Lett.* 282 182–187. 10.1111/j.1574-6968.2008.01119.x 18355287

[B20] Fuerst J. A. (1995). The Planctomycetes: emerging models for microbial ecology, evolution and cell biology. *Microbiology* 141 1493–1506. 10.1099/13500872-141-7-1493 7551018

[B21] Gasol J. M. del Giorgio P. A. Massana R. Duarte C. M. (1995). Active versus inactive bacteria: size-dependence in a coastal marine plankton community. *Mar. Ecol. Prog. Ser.* 128 91–97. 10.3354/meps128091

[B22] Hanson R. S. Hanson T. E. (1996). Methanotrophic bacteria. *Microbiol. Rev.* 60 439–471. 8801441 10.1128/mr.60.2.439-471.1996 PMC239451

[B23] Huber H. Stetter K. O. (2006). *Desulfurococcales. The Prokaryotes* Vol. 3 New York, NY: Springer

[B24] Inskeep W. P. Jay Z. J. Macur R. E. Clingenpeel S. Tenney A. Lovalvo D. (2015). Geomicrobiology of sublacustrine thermal vents in Yellowstone Lake: geochemical controls on microbial community structure and function. *Front. Microbiol.* 6:1044 10.3389/fmicb.2015.01044 PMC4620420 26579074

[B25] Kan J. Clingenpeel S. Macur R. E. Inskeep W. P. Lovalvo D. Varley J. (2011). Archaea in Yellowstone Lake. *ISME J.* 5 1784–1795. 10.1038/ismej.2011.56 21544103 PMC3197168

[B26] Kan J. Wang K. Chen F. (2006). Temporal variation and detection limit of an estuarine bacterioplankton community analyzed by denaturing gradient gel electrophoresis (DGGE). *Aquat. Microb. Ecol.* 42 7–18. 10.3354/ame042007

[B27] Kirchman D. (1983). The production of bacteria attached to particles suspended in a freshwater pond. *Limnol. Oceanogr.* 28 858–872. 10.4319/lo.1983.28.5.0858

[B28] Kirchman D. (2002). The ecology of *Cytophaga*-Flavobacteria in aquatic environments. *FEMS Microbiol. Ecol.* 39 91–100. 10.1111/j.1574-6941.2002.tb00910.x 19709188

[B29] Kirchman D. Mitchell R. (1982). Conribution of particle-bound bacteria to microheterotrophic activity in five coastal ponds and two marshes. *Appl. Environ. Microbiol.* 43 200–209. 16345921 10.1128/aem.43.1.200-209.1982 PMC241801

[B30] Lage O. M. Bondoso J. (2012). Bring Planctomycetes into pure culture. *Front. Microbiol.* 3:405 10.3389/fmicb.2012.00405 PMC3538630 23335915

[B31] Langner H. Jackson C. R. McDermott T. R. Inskeep W. P. (2001). Rapid oxidation of arsenite in a hot spring ecosystem, Yellowstone National Park. *Environ. Sci. Technol.* 35 3302–3309. 10.1021/es0105562 11529568

[B32] Lehours A. Evans P. Bardot C. Joblin K. Gerard F. (2007). Phylogenetic diversity of Archaea and Bacteria in the anoxic zone of a meromictic lake. *Appl. Environ. Microbiol.* 73 2016–2019. 10.1128/AEM.01490-06 17261512 PMC1828810

[B33] Lindstrom E. S. Kamst-Van Agterveld M. P. Zwart G. (2005). Distribution of typical freshwater bacterial groups is associated with pH, temperature, and lake water retention time. *Appl. Environ. Microbiol.* 71 8201–8206. 10.1128/AEM.71.12.8201-8206.2005 16332803 PMC1317352

[B34] Lovalvo D. Clingenpeel S. R. McGinnis S. Macur R. E. Varley J. D. Inskeep W. P. (2010). A geothermal-linked biological oasis in Yellowstone Lake, Yellowstone National Park, Wyoming. *Geobiology* 8 327–336. 10.1111/j.1472-4669.2010.00244.x 20491946

[B35] Macur R. E. Jay Z. J. Taylor W. P. Kozubal M. A. Kocar B. D. Inskeep W. P. (2013). Microbial community structure and sulfur biogeochemistry in mildly-acidic sulfidic geothermal springs in Yellowstone National Park. *Geobiology* 11 86–99. 10.1111/gbi.12015 23231658

[B36] Macur R. E. Langner H. W. Kocar B. D. Inskeep W. P. (2004). Linking geochemical processes with microbial community analysis: successional dynamics in an arsenic-rich, acid-sulphate-chloride geothermal spring. *Geobiology* 2 163–177. 10.1111/j.1472-4677.2004.00032.x

[B37] Margulies M. Egholm M. Altman W. E. Attiya S. Bader J. S. Bemben L. A. (2005). Genome sequencing in microfabricated high-density picolitre reactors. *Nature* 437 376–380. 16056220 10.1038/nature03959 PMC1464427

[B38] Melis A. Happe T. (2001). Hydrogen production. Green algae as a source of energy. *Plant Physiol.* 127 740–774. 10.1104/pp.010498 11706159 PMC1540156

[B39] Morgan L. A. Blackwell D. D. Spafford R. E. Smith R. B. (1977). Heat flow measurements in Yellowstone Lake and the thermal structure of the Yellowstone caldera. *J. Geophys. Res.* 82 3719–3732. 10.1029/JB082i026p03719

[B40] Morgan L. A. Shanks W. C. I. I. I. Lovalvo D. Johnson S. Y. Stephenson W. Pierce K. L. (2003). Exploration and discovery in Yellowstone Lake: results from high-resolution sonar imaging, seismic reflection profiling, and submersible studies. *J. Volcanol. Geotherm. Res.* 122 221–242. 10.1016/S0377-0273(02)00503-6

[B41] Morgan L. A. Shanks W. C. I. I. I. Pierce K. L. Lovalvo D. A. Lee G. K. Webring M. W. (2007). “The floor of Yellowstone Lake is anything but quiet – new discoveries from high resolution sonar imaging, seismic-reflection profiling, and submersible studies,” in *Integrated Geoscience Studies in the Greater Yellowstone Area – Volcanic, Tectonic, and Hydrothermal Processes in the Yellowstone Geoecosystem* ed. Morgan L. A. (Boulder, CO: U.S. Geological Survey Professional Paper 1717) 95–126.

[B43] Pernthaler J. Sattler B. Simek K. Schwarzenbacher A. Psenner R. (1996). Top-down effects on the size-biomass distribution of a freshwater bacterioplankton community. *Aquat. Microbiol. Ecol.* 10 255–263. 10.3354/ame010255

[B44] Rosel S. Allgaier M. Grossart H. P. (2012). Long-term characterization of free-living and particle-associated bacterial communities in Lake Tiefwaren reveals distinct seasonal patterns. *Microbiol. Ecol.* 64 571–583. 10.1007/s00248-012-0049-3 22526401

[B45] Rusch D. B. Halpern A. L. Sutton G. Heidelberg K. B. Williamson S. Yooseph S. (2007). The sorcerer II global ocean sampling expedition: northwest atlantic through eastern tropical Pacific. *PLoS Biol.* 5:e77 10.1371/journal.pbio.0050077 PMC1821060 17355176

[B46] Salcher M. M. Pernthaler J. Posch T. (2010). Spatiotemporal distribution and activity patterns of bacteria from three phylogenetic groups in an oligomesotrophic lake. *Limnol. Oceanogr.* 55 846–856. 10.4319/lo.2009.55.2.0846

[B47] Salcher M. M. Pernthaler J. Zeder M. Psenner R. Posch T. (2008). Spatio-temporal niche separation of planktonic Betaproteobacteria in an oligo-mesotrophic lake. *Environ. Microbiol.* 10 2074–2086. 10.1111/j.1462-2920.2008.01628.x 18430016

[B48] Schullery P. Varley J. D. (1995). “Cutthroat trout and the yellowstone ecosystem,” in *The Yellowstone Lake Crisis: Confronting a Lake Trout Invasion* eds Varley J. D. Schullery P. (Wyoming: A Report to the Director of the National Park Service) 12–21.

[B49] Spear J. R. Walker J. J. McCollom T. M. Pace N. R. (2005). Hydrogen and bioenergetics in the Yellowstone geothermal ecosystem. *Proc. Natl. Acad. Sci. U.S.A.* 102 2555–2560. 10.1073/pnas.0409574102 15671178 PMC548998

[B50] Stevenson L. H. (1978). A case for bacterial dormancy in aquatic systems. *Microbiol. Ecol.* 4 127–133. 10.1007/BF02014283 24231971

[B51] Thomas F. Hehemann J. Rebuffet E. Czjzek M. Michel G. (2011). Environmental and gut Bacteroidetes: the food connection. *Front. Microbiol.* 2:93 10.3389/fmicb.2011.00093 PMC3129010 21747801

[B52] Yang T. Lyons S. Aguilar C. Cuhel R. Teske A. (2011). Microbial communities and chemosynthesis in Yellowstone Lake sublacustrine hydrothermal vent waters. *Front. Microbiol.* 2:130 10.3389/fmicb.2011.00130 PMC3116135 21716640

